# The application and practice of the electronic clinical pathway

**DOI:** 10.1186/1479-5876-10-S2-A57

**Published:** 2012-10-17

**Authors:** Min Zhang

**Affiliations:** 1 Department of Hepatobiliary Surgery, the First Affiliated Hospital of Medical School of Zhejiang University, Hangzhou, 310009, China

## Background

Since the Ministry of Health of the People's Republic of China carried out the clinical pathway in 2009, one hundred and ten pilot hospitals in China have implemented more than 330 sorts of clinical pathways, with some hospitals adopting the hard copy version. With the increasing number of diseases in clinical pathway and the increasing number of cases in clinical practice, as well as the developing informatization reform in hospital, the realization of electronic management of the clinical pathway is urgently needed.

## Methods

With the partition of original time division axis by key diagnosis and treatment nodes, the First Affiliated Hospital of Zhejiang University, College of Medicine has developed the electronic edition of clinical pathways through the integration of the electronic medical records, hospital information system, picture archiving and communication system, laboratory information management system and so on, which were based on the key nodes and gradually put this into clinical practice since March, 2010.

## Result

Ninety five sorts of clinical pathways were developed, with 76 applied into practice. A total of 8,782 cases were managed in the clinical pathway, of which 5,913 successfully completed, with a completion rate of 67.25% and a mutational rate of 29.50%. During the recent three years, with the implementation of clinical path management, the average time length of hospital stay and preoperative hospital stay were decreased, as well as the disease mortality, hospital infection rates, the postoperative complication rates. The average cost by disease and the average daily cost remained flat or turned to be even lower.

## Conclusion

The application of the electronic clinical pathway can effectively improve the efficiency and quality of health care services, and appropriately control the growth of medical cost. Nevertheless, the understanding and compliance of the clinical path application by the medical staffs is to be enhanced further. Meanwhile, the key to the implementation of the clinical pathway is to provide the clinical path management software, which is consistent with the hospital information system and can effectively reduce the workload of medical staffs.

